# High-Energy Concomitant Femoral Shaft Fracture, Tibial Tuberosity Avulsion, and Multiligament Knee Injury Involving the Posterior Cruciate Ligament and Posterolateral Corner in an Adult: A Case Report

**DOI:** 10.7759/cureus.104787

**Published:** 2026-03-06

**Authors:** Keita Akao, Yuki Yamanashi, Kohei Hashimoto, Yoshifumi Ohashi, Nobunori Takahashi

**Affiliations:** 1 Department of Orthopaedics, Aichi Medical University, Nagakute, JPN

**Keywords:** multiligament knee injury, posterior cruciate ligament, posterolateral corner, staged reconstruction, tibial tuberosity avulsion

## Abstract

Adult tibial tuberosity avulsion fractures are rare, and their coexistence with a femoral shaft fracture and combined posterior cruciate ligament (PCL) and posterolateral corner (PLC) injury is exceptional. Such constellations can obscure knee instability at presentation and complicate tunnel placement if early ligament reconstruction is attempted.

A 37-year-old man sustained a high-energy traffic injury with a right femoral shaft fracture and an isolated tibial tuberosity avulsion. MRI and stress testing revealed a multiligament knee injury involving the PCL and PLC, including a repairable distal lateral collateral ligament (LCL) avulsion and a lateral meniscal (LM) tear. On hospital day 2, stage one included intramedullary nailing of the femur, fixation of the tuberosity with two 4.5-mm cannulated cancellous screws and washers, and limited PLC repair/augmentation (reattachment of the distal LCL avulsion with an anchor and LM suturing). At five months, with the union of the tuberosity confirmed, residual posterior sag (6-mm side-to-side) persisted while varus/rotational laxity was minimal. Stage two comprised single-bundle arthroscopic PCL reconstruction using a semitendinosus-gracilis autograft (femoral EndoButton; Smith & Nephew, London, UK), tibial double-spike plate; 50 N at 90°) after removal of the tibial screws to avoid tunnel-implant collision. At 18 months after the initial operation (12 months after PCL reconstruction), knee stability in flexion was restored with an approximately 3-mm gravity-sag difference. Femoral union was achieved. Knee Injury and Osteoarthritis Outcome Score (KOOS) subscales were as follows: Symptoms 85.7, Pain 83.3, Activities of Daily Living 91.2, Sports 75.0, and Quality of Life 62.5; the Tegner activity level improved from 7 preinjury to 6 at final follow-up.

Early bony stabilization with limited acute PLC repair provided bridging stability and preserved options for accurate tunnel placement. Delayed, targeted PCL reconstruction addressed the dominant posterior instability while mitigating risks of arthrofibrosis and hardware-tunnel interference. In complex high-energy injuries with concomitant fractures, a staged and individualized approach, prioritizing bony union, reassessing instability, and reconstructing only the dominant deficient ligament, can yield favorable functional outcomes.

## Introduction

Adult tibial tuberosity avulsion fractures are extremely uncommon because the proximal tibial apophysis has fused in skeletally mature patients, and in adults, the extensor mechanism more commonly fails at the patellar tendon or patella itself, where the bone-tendon interface is relatively stronger than the apophyseal region seen in adolescents. In epidemiologic reports largely derived from adolescents, tibial tubercle fractures account for ~3% of proximal tibial fractures and <1% of physeal injuries in pediatric and adolescent populations, where these injuries represent apophyseal (physeal-related) avulsion patterns [1,2]. Robust adult incidence data are lacking; however, a CT-based series of adult proximal tibial fractures noted tibial tubercle involvement in ~11%, although most of these cases represented extension of tibial plateau fractures rather than isolated extensor-mechanism avulsions [3]. These injuries are often associated with high-energy trauma and may coexist with additional knee injuries that are easily overlooked in the acute setting. To our knowledge, the simultaneous occurrence of a femoral shaft fracture, an isolated tibial tuberosity avulsion (without tibial plateau involvement), and combined posterior cruciate ligament (PCL) and posterolateral corner (PLC) injury involving the lateral collateral ligament (LCL) in the same limb of an adult has not been previously reported. This constellation poses unique diagnostic and therapeutic challenges. Pain, swelling, and the priority of early fracture stabilization can mask knee instability, while bony hardware may interfere with tunnel placement if ligament reconstruction is attempted too early. A staged approach that begins with initial bony stabilization and primary PLC repair and is followed by delayed PCL reconstruction after soft-tissue recovery and tubercle union can mitigate these issues and has been advocated in the management of multiligament knee injuries [4,5]. We present a case of combined tibial tuberosity avulsion, femoral shaft fracture, and multiligament knee injury involving the PCL and PLC that was successfully managed with a planned two-stage surgical approach. The aim of this report is to emphasize the importance of accurately recognizing this rare injury pattern to avoid missed diagnoses, thereby enabling appropriate staged surgical planning and achieving favorable functional outcomes.

An abstract of this study was previously presented in poster format at the 147^th^ Meeting of the Central Japan Association of Orthopaedic Surgery and Traumatology, held on October 3-4, 2025.

## Case presentation

History of present trauma

**Figure 1:**
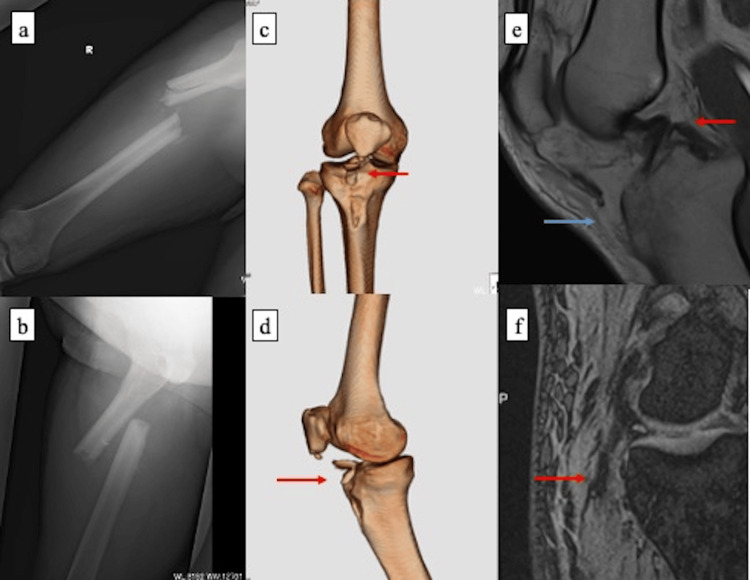
Preoperative radiographic, CT, and MRI findings (a-b) Anteroposterior and lateral radiographs of the right femur demonstrating a femoral shaft fracture. (c-d) CT images of the right knee demonstrating an avulsion fracture of the tibial tuberosity (red arrows). (e) MRI of the right knee demonstrating a posterior cruciate ligament injury (red arrow) and a tibial tuberosity avulsion fracture (blue arrow). (f) MRI demonstrating a lateral collateral ligament injury (red arrow).

A 37-year-old man was admitted to our emergency department after a traffic collision between a car and a motorcycle. He was the driver at the time of the accident, and his right knee was reportedly in a flexed position. Given the presence of a contusion over the anterior aspect of the proximal tibia, a dashboard-type mechanism was suspected. On examination, he had right thigh pain and deformity, right knee pain, and contusion of the lower leg. Radiographs demonstrated a right femoral shaft fracture and an avulsion fracture of the tibial tuberosity. For damage control, external fixation of the femoral shaft was performed the same day (Figures 1a-1d). On hospital day 1, a knee MRI was obtained, and an orthopedic knee consultation was requested. The MRI revealed a complex ligamentous injury. Additional varus stress testing confirmed instability, leading to a diagnosis of a multiligament knee injury involving the PCL and PLC, including the LCL, with an associated lateral meniscal (LM) tear (Figures 1e-1f).

First stage of the operation

**Figure 2:**
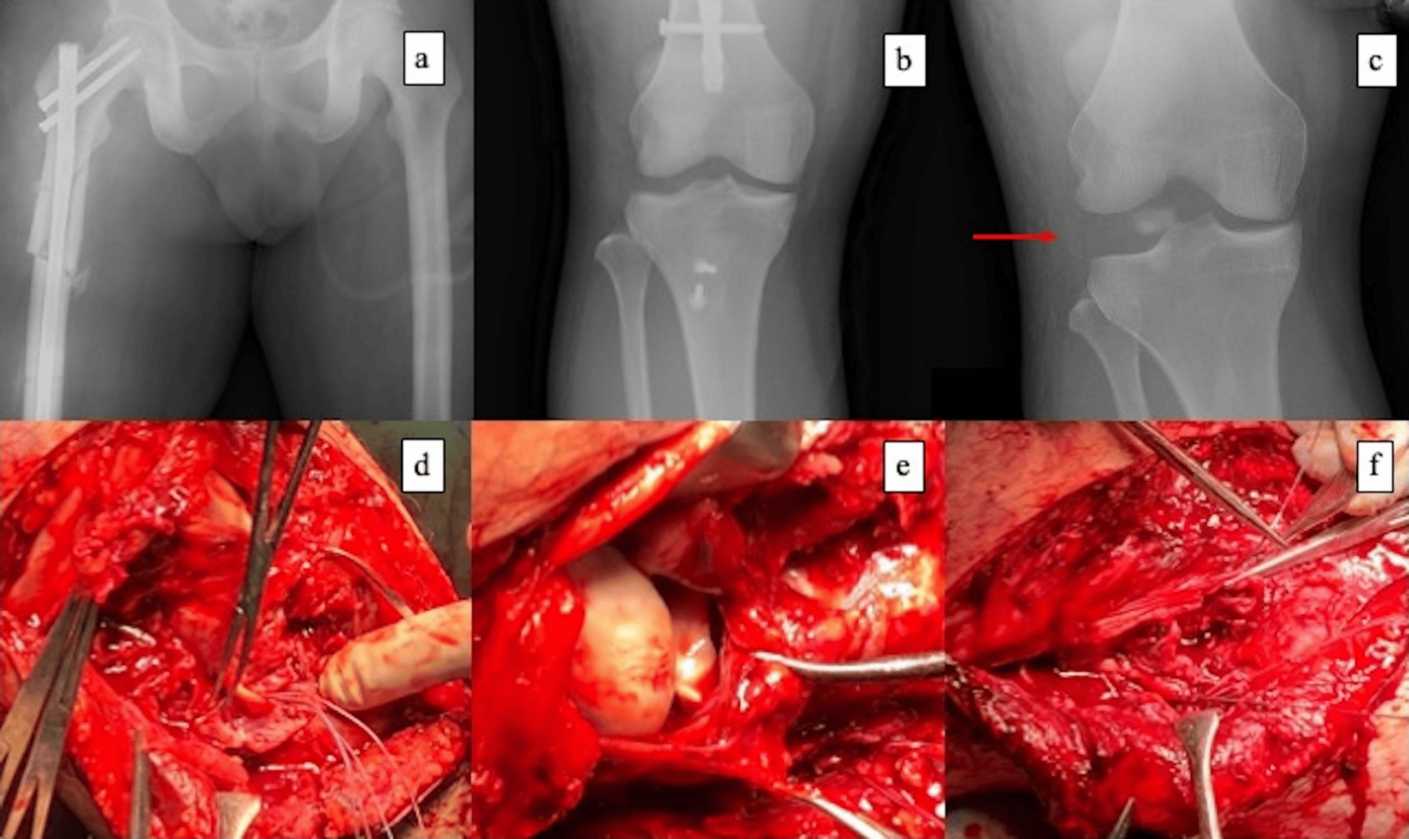
First-stage surgery: perioperative radiographs and intraoperative findings (a-b) Postoperative radiographs showing femoral shaft fixation with an intramedullary nail and tibial tuberosity fixation with cannulated cancellous screws. (c) Preoperative varus stress radiograph demonstrating grade III lateral opening, consistent with severe lateral-sided instability. (d-f) Intraoperative findings and repair; The lateral collateral ligament was torn near its fibular insertion and was repaired using a suture anchor placed in the fibular head. A longitudinal tear of the lateral meniscus (midbody) was repaired under direct visualization. The lateral capsule was then repaired.

On hospital day 2, the first-stage operation was performed. The femoral shaft fracture was treated with intramedullary nailing on a traction table using a Femoral Recon Nail (DePuy Synthes, Raynham, MA, USA). The patient was then placed supine. A 15-cm oblique incision was made on the lateral aspect of the knee, followed by dissection of the iliotibial band and joint capsule to expose the fracture site. The tibial tuberosity avulsion was fixed with two 4.5-mm cannulated cancellous screws (CCS) and washers. For acute PLC management, we performed limited repair/augmentation to enhance early stability, including reattachment of a repairable distal LCL avulsion using an anchor inserted into the fibular head. The LM was sutured under direct visualization, and the joint capsule was then repaired, completing the procedure. Intraoperatively, the PLC pattern was focal and repairable (distal LCL avulsion with a repairable LM tear) rather than an irreparable midsubstance disruption, supporting an acute repair strategy (Figures 2a-2f)

Postoperative rehabilitation proceeded as follows. For the first two weeks after surgery, the knee was immobilized with a brace, and the patient was maintained non-weight bearing. Partial weight bearing and range-of-motion (ROM) exercises were started two weeks postoperatively. Knee flexion was limited to 90° for the first four weeks. Full weight bearing and flexion beyond 120° were permitted with a hinged hard brace beginning six weeks postoperatively.

Second stage of the operation

**Figure 3:**
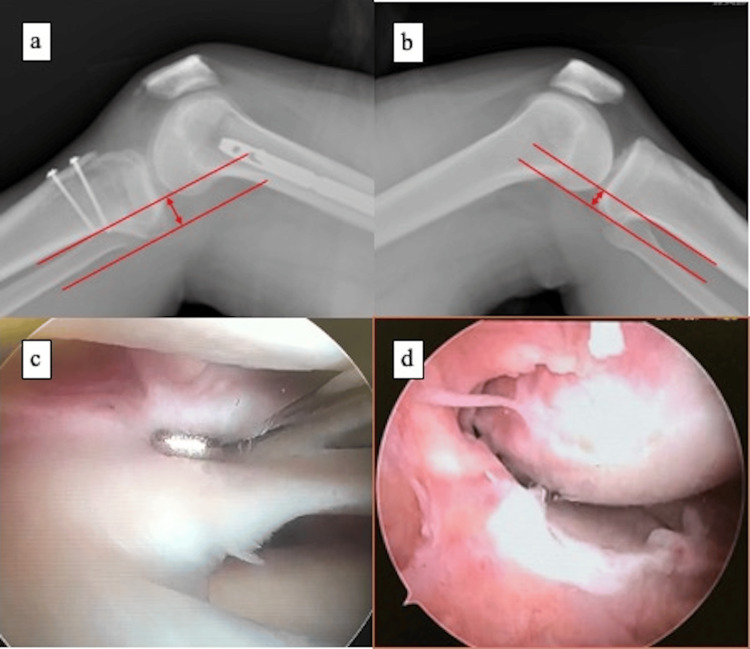
Pre-second-stage radiographs and intraoperative findings (a-b) Gravity-sag views demonstrating a 6-mm side-to-side difference between the affected and unaffected knees. Dashed lines indicate the posterior cortical reference lines used to measure posterior tibial translation, and the double-headed arrow indicates the measured side-to-side difference. (c) Arthroscopic confirmation of healing of the repaired lateral meniscus. (d) Arthroscopic visualization of posterior cruciate ligament injury.

At five months after the initial surgery, plain radiographs and CT demonstrated a solid union of the tibial tuberosity. However, knee instability in flexion persisted, and a gravity-sag view demonstrated a 6-mm side-to-side difference (Figures 3a-3d). During the second-stage operation, the two CCSs inserted during the first surgery were removed to allow adequate tunnel placement for PCL reconstruction. Arthroscopic single-bundle PCL reconstruction using the semitendinosus and gracilis tendons was then performed. The femoral tunnel was created at the anatomic PCL footprint via the anteromedial portal, and the tibial tunnel was drilled using a PCL guide. The graft was fixed with an EndoButton (Smith & Nephew, London, UK) on the femoral side and a double-spike plate on the tibial side under 50 N of tension with the knee flexed at 90°. During arthroscopy, complete healing of the LM was also confirmed.

Partial weight bearing and ROM exercises were initiated two weeks after reconstruction, with weight bearing and ROM advanced weekly as tolerated.

Postoperative outcomes and follow-up

**Figure 4:**
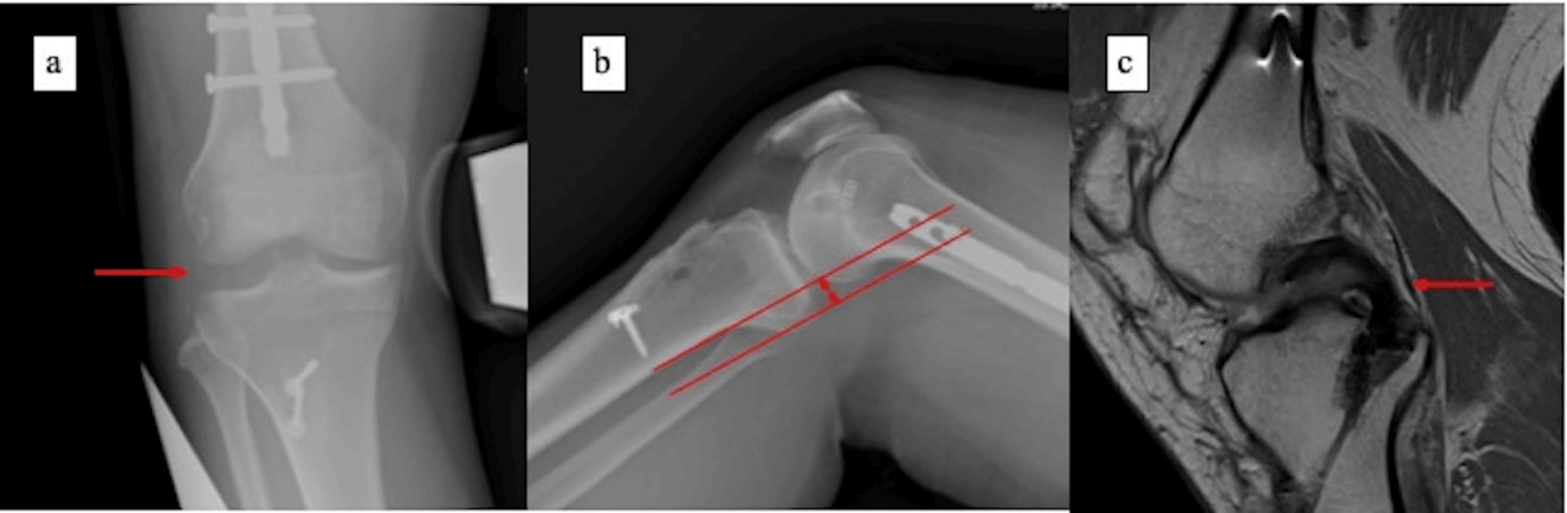
Final imaging findings (a) Varus stress radiographs at final follow-up showing improved lateral stability compared with the preoperative status. (b) Gravity-sag views demonstrating an approximately 3-mm side-to-side difference between the affected and unaffected knees. (c) MRI at final follow-up confirming good graft/ligament continuity of the posterior cruciate ligament.

At 18 months after the initial operation (12 months after PCL reconstruction), the gravity-sag view demonstrated an approximately 3-mm side-to-side difference, and knee stability in flexion had been restored (Figures 4a-4c). Complete union of the femoral shaft fracture was also achieved. Knee injury and Osteoarthritis Outcome Score (KOOS) subscales were as follows: Symptoms 85.7, Pain 83.3, Activities of Daily Living 91.2, Sports 75.0, and Quality of Life 62.5, indicating good recovery in pain and daily function, with moderate residual limitation in sports activity and quality of life [6]. The Tegner activity level improved from 7 pre-injury to 6 at final follow-up [7].

## Discussion

In this report, we presented a relatively rare trauma case of simultaneous tibial tuberosity avulsion, femoral shaft fracture, and multiligament knee injury involving the PCL and PLC. A favorable outcome was achieved by early recognition of these injuries, timely intervention, and a staged surgical strategy. The usual mechanisms of avulsion of the tibial tuberosity are (1) sudden quadriceps contraction during knee extension and (2) passive knee flexion occurring against a rapidly contracting quadriceps [8]. These mechanisms are most commonly described in adolescents. In adults, tibial tuberosity avulsions are typically associated with high-energy trauma. A PCL injury is similarly attributed to a posteriorly directed force applied to the anterior aspect of the proximal tibia [9,10]. In the present case, given the flexed knee position at the time of injury and contusion over the anterior proximal tibia, a dashboard-type posteriorly directed force was considered the most plausible mechanism, whereby posterior tibial translation may have contributed to PCL disruption and the associated injury pattern. Adult tibial tuberosity fractures are extremely uncommon, and, to the best of our knowledge, the specific combination of associated injuries seen in our patient has not been previously reported in an adult. Nevertheless, in the setting of a comparable mechanism of injury, clinicians should maintain a high index of suspicion for this entity at the time of diagnosis. Surgical treatment for PCL injuries is broadly categorized into repair and reconstruction. Recent expert consensus indicates that isolated repair is not recommended for complete grade III midsubstance PCL injuries, for which anatomic reconstruction is preferred; conversely, repair may be considered in the acute setting when the injury involves an isolated bony or focal soft-tissue avulsion confined to a single stabilizing structure [4,11,12]. In our case, the PLC injury pattern included a repairable distal LCL avulsion, representing a focal component of the PLC, aligning with consensus indications for acute repair, whereas midsubstance grade III tears would warrant anatomic reconstruction. Furthermore, case series of multiligament knee injuries associated with dislocation have reported favorable outcomes with a staged strategy: early treatment of the medial and lateral complexes, followed by delayed ACL/PCL reconstruction based on residual instability after recovery of range of motion [4,5,13].

In the present case, a femoral shaft fracture and tibial tuberosity avulsion fracture necessitated prioritizing bony union. Early ligament reconstruction was additionally deferred because of the risk of tunnel-implant collision with the femoral nail and tibial screws. During the acute phase, we performed limited repair/augmentation of the PLC to provide bridging stability. After bony union and soft-tissue recovery, reevaluation showed persistent posterior sag with only minimal varus/rotational laxity; therefore, isolated PCL reconstruction was undertaken. This approach yielded adequate PLC stability with repair alone, while the two-stage PCL reconstruction corrected the posterior instability, resulting in a favorable functional outcome. Given the heterogeneity of severe trauma, high-quality evidence remains limited; thus, a stepwise, individualized strategy that considers injury pattern (midsubstance vs. avulsion), associated fractures, surgical invasiveness, and potential tunnel-implant interference is warranted in similar complex injuries.

This is a single-case report without a comparator. Contemporary PLC reconstruction techniques may differ from those used historically. Long-term outcomes beyond 18 months remain unknown.

## Conclusions

Adult tibial tuberosity avulsion fractures are rare, and their coexistence with a femoral shaft fracture and combined PCL and PLC injury is exceptional. In our patient, early bony stabilization with limited acute PLC repair followed by delayed isolated PCL reconstruction after tubercle union restored stability and function. This staged, individualized strategy mitigated the risks of tunnel-implant collision and arthrofibrosis while targeting the dominant posterior instability. Clinicians should maintain a high index of suspicion for PCL injury resulting from a dashboard-type posteriorly directed force, as well as associated PLC injury, in similar high-energy mechanisms and tailor the timing and extent of surgery to fracture pattern, soft-tissue condition, and concomitant injuries.
